# The Chronic Insane

**Published:** 1867-06

**Authors:** 


### THE CHRONIC INSANE.

Dr. Lee, of New York, presented the following:—

*Resolved*, That providing for the poor chronic insane in the jails and almshouses of our country, as at present practiced in nearly all the States of the Union, is a gross violation of the laws of humanity, and contrary to the divine injunction of doing to others as we would be done by.

*Resolved*, That when the regular hospitals for the insane of a State are insufficient to accommodate both acute and chronic cases that are sent to them, this Association would strongly recommend the procurement of a suitable amount of land in the vicinity, and the erection of convenient, well-planned, and well-ventilated, but comparatively inexpensive buildings, in connection with and under the same general supervisor as the hospitals themselves, where those who are able to labor, and would be benefited by light, regulated employment, may be suitably accommodated and properly cared for.

*Resolved*, That the example of Mass. in establishing asylums for the accommodation and humane treatment of the chronic insane, is worthy of all praise and imitation, and in the opinion of this Association, such institutions, if rightly inaugurated and judiciously carried on, will be a benefit to the State in an economical point of view, will raise the character of the State hospitals, and will greatly subserve the interests of the insane generally.

*Resolved*, That as the present insane hospitals are capable of accommodating but a small proportion of the 40,000 of the insane of the United States, and as almshouse and jail provision is not adapted to their proper care and treatment, this Association would recommend to the proper State authorities to make such further provision in the direction above indicated, as may tend to the amelioration of their condition, if not the restoration of their rational and moral faculties.

They were referred to the following committee, *viz.*:—Drs. C. A. Lee, New York; Gundry, of Ohio; John Fonerton Walker, of Massachusetts; and Chipley, of Kentucky.

The Association then adjourned, *sine die.*

